# CT导引下^32^P-磷酸铬-聚-L-乳酸粒子植入治疗兔VX2肺肿瘤的实验研究

**DOI:** 10.3779/j.issn.1009-3419.2011.01.01

**Published:** 2011-01-20

**Authors:** 栋辉 潘, 敏 杨, 宇平 徐, 立振 王, 璐 刘, 培林 黄

**Affiliations:** 1 214063 无锡，江苏省原子医学研究所，卫生部核医学重点实验室，江苏省分子核医学重点实验室 Jiangsu Key Laboratory of Molecular Nuclear Medicine, Key Laboratory of Nuclear Medicine, Ministry of Health, Jiangsu Institute of Nuclear Medicine, Nanjing 214063, China; 2 210009 南京，东南大学核医学技术研究所 Nuclear Medicine Institute of Southeast University, Nanjing 210009, China

**Keywords:** 肺肿瘤, ^18^F-脱氧葡萄糖, PET/CT, ^32^P-CP-PLLA粒子, 兔, Lung neoplasms, ^18^F-FDG, PET/CT, ^32^P-CP-PLLA microparticle, Rabbit

## Abstract

**背景与目的:**

新型放射性植入剂^32^P-磷酸铬-聚-L-乳酸（^32^P-CP-PLLA）粒子具有良好的生物相容性和降解性，适用于实体肿瘤的近距离放射治疗。本研究旨在探讨兔VX2肺肿瘤经^32^P-CP-PLLA粒子瘤体间植入近距离治疗前后PET/CT显像及病理学的变化，分析^32^P-CP-PLLA粒子植入对荷VX2肺癌兔肿瘤生长及凋亡相关蛋白的影响。

**方法:**

24只荷瘤兔随机分成4组。每组6只。1组-3组为治疗组；4组为对照组。在CT导引下经皮穿刺将总放射性活度为93 MBq、185 MBq和370 MBq的^32^P-CP-PLLA粒子分别植入1组、2组和3组肿瘤组织内。对照组不做任何干预。分别在治疗后第0天、第3天、第7天和第14天进行^18^F-FDG PET/CT显像，观察标准摄取值（standardized uptake value, SUV）的变化。最后1次PET/CT显像后处死荷瘤兔，取出肿瘤组织，进行病理学检查和免疫组织化学分析，比较肿瘤细胞形态和凋亡基因（*bcl-2*, *bax*）表达的变化。

**结果:**

第0天时，治疗组和对照组之间SUVmax无明显差异。治疗后第14天，1组、2组和3组SUVmax值分别为1.1±0.19、0.80±0.10和2.85±0.15，均较对照组（5.61±0.50）明显下降。第7天-第14天时，1组和2组SUVmax较第3天呈现持续下降趋势，且呈剂量效应关系（*P* < 0.05）。治疗后第3天-第14天，3组SUVmax较第0天显著上升，并在第7天达到峰值，后明显下降。同期3组SUVmax明显低于对照组SUVmax。HE染色显示近粒子处的肿瘤细胞变性坏死，坏死程度随剂量的增加而严重。3组可见坏死组织周围有大量炎性细胞浸润，而1组-2组炎性细胞浸润不明显。免疫组化显示治疗组bcl-2表达强度低于对照组，bax表达强度高于对照组（*P* < 0.05）。治疗组bcl-2/bax比值明显下调（*P* < 0.05）。凋亡基因的表达呈剂量效应关系。

**结论:**

^32^P-CP-PLLA粒子持续照射可直接杀伤VX2肿瘤细胞从而抑制其葡萄糖代谢功能。远离粒子处虽可见存活肿瘤细胞，但凋亡基因表达明显异于对照组。^32^P-CP-PLLA粒子可通过电离辐射诱导*bcl-2*和*bax*基因参与VX2移植瘤细胞凋亡过程的调控，从而抑制肿瘤生长。

## Abstract (en)

**Background and objective:**

^32^P-chromic phosphate-poly (L-lactic) acid (^32^P-CP-PLLA) microparticle is a novel potent brachytherapy implant, which has good biocompatibility and biodegradability. The aim of this study is to investigate the changes of pathology and PET/CT images in VX2 rabbit tumor afer treatment with intratumorol administration of ^32^P-CP-PLLA microparticles, and to explore the effects and influence of tumor growth and apoptosis related proteins in VX2 lung tumor treatment with ^32^P-CP-PLLA microparticles.

**Methods:**

Twenty-four tumor bearing rabbits were randomly divided into 4 groups (6 in each group). Group 1, 2 and 3 were treated groups; group 4 was the control. Under CT guidance, ^32^P-CPPLLA microparticles were implanted into tumors. Low, medium and high treatment doses were 93 MBq (group 1), 185 MBq (group 2) and 370 MBq (group 3), respectively. ^18^F-FDG PET/CT was performed at d0, d3, d7 and d14 afer intratumoral administration. In the control group, ^18^F-FDG PET/CT images were acquired at the same time points but without treatment. The standardized uptake value (SUV) of tumor regions were calculated. Afer last PET/CT imaging, the rabbits were euthanized and the tumors were removed for histological and immunohistochemical examination. The pathology and the expression of apoptosis related proteins (bcl-2, bax) were compared.

**Results:**

No signifcant difference of SUVmax was observed between the treatment groups and the control group at d0. At d14, the SUVmax values for group 1, 2 and 3 were 0.80±0.10, 1.1±0.19 and 2.85 ±0.15, respectively, and were signifcantly lower than that of the control group (5.61±0.50)(*P* < 0.05). Signifcant dose-response relationship was observed in SUVmax between group 1 and group 2, and the SUV values gradually decreased from d7 to d14 afer treatment. In group 3, SUVmax gradually increased and reached a peak at d7 then signifcantly decreased. The SUVmax values of group 3 were signifcantly lower than those of the control at the same time point (*P* < 0.05). HE staining found degenerative necrosis at the site was nearby the microparticle. Necrosis became serious increasing with the radioactivity. Inflammatory cell infltration was rarely seen in tumors treated with 93 MBq or 185 MBq ^32^P-CP-PLLA microparticles. In contrast, the necrotic area was surrounded by marked inflammatory cell infltration in group 3. IHC analysis showed that the expression of bcl-2 in treated groups were lower than those in the control group, and the expression of bax in treated group was higher than those in the control group (*P* < 0.05). The ratio of bcl-2/bax protein signifcantly decreased in the treated group (*P* < 0.05). Dose dependence was seen in the expression of apoptosis related proteins.

**Conclusion:**

The sustained irradiation of ^32^P-CP-PLLA microparticles can direct kill the VX2 tumor cell, thus the glycolysis of which were suppressed. Although the alive tumor cells still presented faraway from the microparticle, the expression of apoptosis related proteins in which were signifcantly different from the control. *Bcl-2* and *bax* gene were induced to participate in regulation for the apoptosis of VX2 tumor cell by ionizing radiation from ^32^P-CP-PLLA microparticles, so that the tumor growth was inhibited.

手术切除、化疗和外放疗是肿瘤治疗的主要手段，但存在着复发率高、治疗效果不理想和毒副作用大等缺陷^[1]^。目前，载有放射性核素如^32^P、^90^Y、^166^Ho、^188^Re和^32^P等的药物内照射治疗恶性实体瘤已经取得较好的成效^[2-5]^。这些放射性药物能直接作用于靶点，同时对正常组织影响较小。相对于其它β射线发射核素，^32^P价廉易得且物理学性能良好。间质注射胶体^32^P-磷酸铬（^32^P-CP）在治疗肺癌、乳腺癌和脑肿瘤等恶性实体瘤方面有一定的应用价值^[6-8]^。但研究^[9]^发现，随着^32^P-CP间质给药剂量的加大，全身药物分布亦增加，对正常组织如肝、脾等的毒性也加大。因此，寻找一种靶向定位更佳的载体形式成为放射性核素内照射治疗研究的重点。聚L-乳酸（poly l-lactic acid, PLLA）是一种生物相容性良好的高分子材料，作为药用辅料被广泛应用于药物缓释系统^[10]^。先前研究^[11]^表明，由^32^P-CP和PLLA混合制备成的^32^P-磷酸铬-聚-L-乳酸（^32^P-CP-PLLA）粒子锚定在植入点，稳定性良好。

VX2鳞癌细胞株是由Shoppe病毒诱发的乳头状瘤恶变后经兔传代而取得的实验性肿瘤。兔VX2肿瘤具有易于移植、侵袭性强、成功率高的特点，其形态学和生物学特性与人类肿瘤相似，并且生长周期短，非常适合实验研究^[12]^。近10年肺癌发病率和死亡率均有明显增高趋势。本研究以荷VX2肺癌兔为模型，采用^18^F-FDG PET/CT显像及病理学研究对^32^P-CP-PLLA粒子瘤体间植入近距离治疗的疗效进行了评价。

## 1 材料和方法

### 1.1 实验动物

新西兰大白兔，4月龄-5月龄，2.0 kg-3.0 kg，雌雄不限，由南京安立默科技有限公司提供。

### 1.2 实验器材与药品

^32^P-CP-PLLA粒子由南京医科大学附属南京第一医院临床核医学中心提供^[13]^。^32^P-CP-PLLA粒子为淡绿色圆柱体，无菌，活度为93 MBq/枚。bcl-2和bax免疫组织化学检测试剂盒购自福州迈新生物技术有限公司。

### 1.3 麻醉方法

采用25%乌拉坦麻醉，剂量为1 g/kg，腹腔注射。5 min后达到麻醉效果，此时兔处于昏睡状态，对手术刺激无明显反应。

### 1.4 模型制作方法

将带有VX2肿瘤的荷瘤兔（上海交通大学实验动物中心赠予）麻醉后，无菌条件下取出肿瘤，置于生理盐水中。在超净台用眼科剪剪成1 mm^3^小瘤块备用。

将正常新西兰大白兔麻醉后，仰卧位固定于自制手术木架，置于CT机床上。左胸部剪毛。取右胸第4-5肋间肩胛骨前缘为穿刺点，消毒穿刺点。将瘤块置入18 G穿刺针中，瘤块的前后均放置0.5 cm长无菌明胶海绵条。在CT引导下刺入左下肺，用针芯推入肺组织中拔针。手术结束，CT扫描肺部，无明显的气胸发生。术后3天内实验兔注射青霉素60万IU/只。3周后行CT检查，当肿瘤直径为10 mm左右，即得荷VX2肺癌兔模型。

### 1.5 ^32^P-CP-PLLA粒子瘤体间植入近距离治疗

24只荷瘤兔随机分成4组，每组6只，雌雄各半。荷瘤兔麻醉后，以仰卧位固定于机床上。在CT导引下经皮穿刺分别将1枚、2枚和4枚^32^P-CP-PLLA粒子分别植入1组、2组和3组荷瘤兔肺部肿瘤组织内，各组总放射性活度分别为93 MBq、185 MBq和370 MBq。4组荷瘤兔为空白对照组，不作任何干预。

### 1.6 PET/CT显像

仪器为美国GE公司Discovery LE型。荷瘤兔PET/CT显像前至少禁食6 h以上。荷瘤兔麻醉后仰卧位固定于机床上。耳缘静脉注射^18^F-FDG 37 MBq/kg（南京安迪科正电子研究发展有限公司提供）55 min-60 min后行^18^F-FDG/PET检查。先行CT透射扫描，然后进行PET发射扫描。经衰减校正和迭代重建后获得CT、PET及两者融合图像。

### 1.7 标准摄取值（standardized uptake value, SUV）检测

利用Xeleris图像融合工作站调用PET/CT图像，获得横断面数据，利用CT图像准确获取肿瘤最大切面，在PET图像上勾画肿瘤边缘，取放射性浓聚比较均匀的部位进行SUV检测，获得肿瘤组织最大SUV（SUVmax）。1组-4组分别在治疗后第0天（治疗当天）、第3天、第7天和第14天进行PET/CT显像。

### 1.8 病理学检查

第14天PET显像后处死大白兔，取出肿瘤组织，质量分数为10%的福尔马林固定，石蜡包埋。进行常规HE染色，观察肿瘤组织的形态学变化。采用SP法检测凋亡相关蛋白bcl-2和bax的表达。bcl-2和bax阳性表达均表现为细胞浆内棕黄色颗粒沉淀。每张切片随机选取6个高倍视野（×400），每个视野计数200个细胞。bcl-2和bax的表达强度以视野内阳性细胞占细胞总数的百分率（%）表示，并取平均值。bcl-2/bax比值根据bcl-2和bax百分率计算。

### 1.9 统计学处理

采用SPSS 10.0统计软件，数据采用Mean±SD表示，采用单因素方差分析进行组间比较。以*P* < 0.05为差异有统计学意义。

## 2 结果

### 2.1 PET/CT表现

^32^P-CP-PLLA粒子瘤体间植入近距离治疗前后治疗区组织的PET显像如图 1所示。第0天时，治疗组和对照组之间SUVmax无明显差异（图 2）。治疗后第14天，治疗组放射性浓聚较对照组明显减少。1组、2组和3组SUVmax值分别为1.1±0.19、0.80±0.10和2.85±0.15，均较对照组（5.61±0.50）明显下降（*P* < 0.05）。第7天-第14天时，1组和2组SUVmax较第3天呈持续下降趋势，且呈剂量效应关系。治疗后第3天-第14天，3组SUVmax较第0天明显上升，并在第7天达到峰值，后明显下降。同期3组SUVmax明显低于对照组。

**1:**
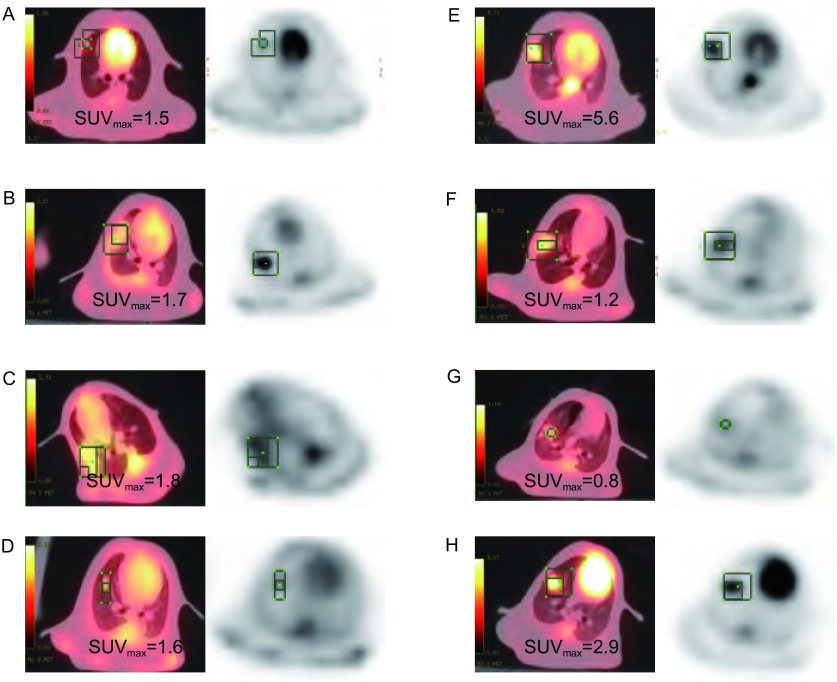
兔VX2肺癌PET/CT显像图。A、B、C和D分别为对照组、1组、2组和3组治疗第0天时PET/CT显像图。E、F、G和H分别为对照组、1组、2组和3组治疗第14天时PET/CT显像图。 PET/CT images of rabbit VX2 lung tumor. A, B, C and D are the PET/CT images of rabbit VX2 lung tumor of the control, group 1, group 2 and group 3 at d0. E, F, G and H are the PET/CT images of rabbit VX2 lung tumor of the control, group 1, group 2 and group 3 at d14 after treatment respectively.

**2:**
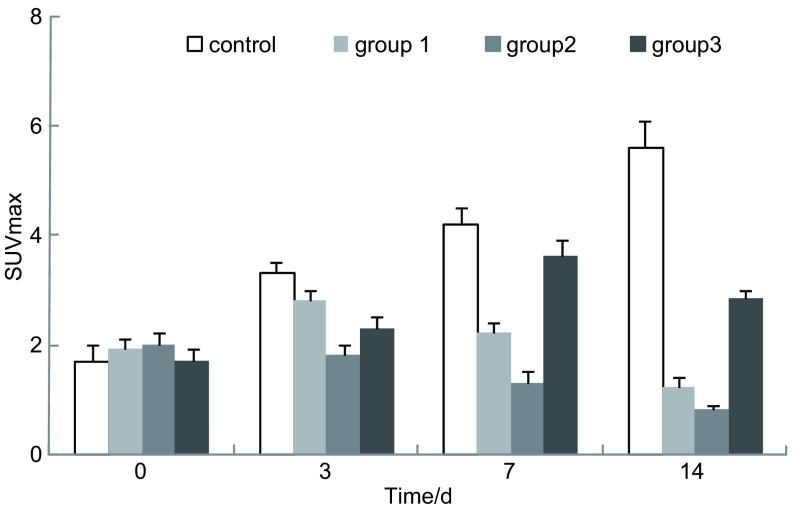
^32^P-CP-PLLA粒子植入后兔VX2肺癌最大标准摄取值（SUVmax）变化图 Changes in the SUVmax of rabbit VX2 lung tumor after implanted with ^32^P-CP-PLLA microparticle

### 2.2 形态学观察

治疗后第14天，病理切片显示近粒子处的肿瘤细胞核缩小，部分破裂溶解，胞浆出现空泡，有均质红染的坏死区域。坏死程度随剂量的增加而严重，但远离粒子处仍可见存活肿瘤细胞。3组可见坏死组织周围有大量炎性细胞浸润，而1组-2组炎性细胞浸润不明显。对照组肿瘤细胞排列密集，核大，深染，瘤巨细胞多见，无明显坏死和炎症（图 3A）。

**3:**
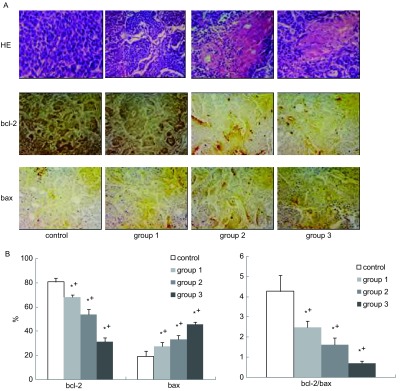
瘤体植入粒子后第14天肿瘤病理切片图。A：第14天后，1组、2组、3组和4组HE染色图及bcl-2和bax的免疫组化图片；B：bcl-2和bax定量分析图。^*^：与对照组比较，*P* < 0.05；^+^各治疗组之间比较，*P* < 0.05。 Histology and immunohistochemistry. A: Tumor stained with HE, bcl-2 and bax of the control, group 1, group 2 and group 3 on d14 after treatment respectively; B: Quantitative assessment of bcl-2 and bax expression. ^*^: *P* < 0.05, bcl-2 and bax expressions of treated group was significantly different than that of the control group; ^+^: *P* < 0.05. Significant differences were seen in bcl-2 and bax expression between each treated groups.

### 2.3 免疫组织化学检测结果

治疗第14天后，1组-4组bcl-2和bax的表达强度及bcl-2/bax比值如图 3B所示。治疗组bcl-2表达强度和bcl-2/bax比值均明显低于对照组，而bax的表达强度均明显高于对照组（*P* < 0.05）。bcl-2和bax的表达强度及bcl-2/bax比值均与剂量明显相关。

## 3 讨论

放射性药物内照射治疗实体瘤疗效已得到证实。其方法是将放射源置于病变部位或其周围，放射源发出射线，其在有效射程内使肿瘤组织发生血管萎缩、细胞凋亡或坏死^[11]^。目前，内照射治疗已用于恶性实体瘤如肝癌、肺癌和胰腺癌等的治疗，效果良好。^32^P是理想的治疗用放射性核素。^32^P发射纯β射线，其最大能量为1.7 MeV，最大组织内射程为8 mm，物理半衰期为14.3天^[14]^。先前研究^[11]^表明^32^P-CP-PLLA粒子将能量绝大部分释放在植入点，对其它非植入脏器影响较低。与胶体^32^P-CP相比，^32^P-CP-PLLA粒子有效地提高了使用的安全性。

新西兰大白兔具有性格温顺、便于试验操作等优点。VX2移植瘤属于鳞癌，种植后易成活^[15]^。本研究以荷VX2肺癌兔为模型，观察了^32^P-CP-PLLA粒子植入对VX2肺癌的治疗效果。

由于治疗后肿瘤的形态改变不会立即显现，且肿瘤中包含的非活性物质（例如坏死）、受损的细胞会使诊断发生困难，因此传统解剖学影像检查（CT或MRI）可能不适于早期疗效监测^[16]^。

与CT相比，^18^F-FDG/PET在临床上疗效评价中有一定的优势。恶性肿瘤细胞的异常增殖需要葡萄糖的过度利用。葡萄糖代谢显像正是利用了肿瘤这种代谢特点。^18^F-FDG是葡萄糖的类似物，静脉注射后经PET显像可灵敏检测出与肿瘤细胞活性相关的异常葡萄糖代谢。临床上，^18^F-FDG PET显像可以有效地监测肿瘤放化疗的疗效^[17]^。

第14天时，治疗组的SUVmax值明显低于对照组，表明^32^P-CP-PLLA粒子瘤体间植入近距离治疗降低了肿瘤细胞的活性从而抑制了其葡萄糖代谢功能，这与病理学观察的结果相一致。^18^F-FDG/PET显示治疗后第7天-第14天，1组和2组的SUVmax较第3天明显下降，且存在剂量效应关系。该效应关系与病理学分析结果相一致，提示^18^F-FDG/PET显像能监测中、低剂量（93 MBq-185 MBq）粒子内照射治疗后肿瘤组织的变化。形态学观察表明，治疗第14天后，3组坏死肿瘤组织周围有大量炎性细胞浸润。由于^18^F-FDG/PET不能区分炎性和增殖肿瘤细胞引起的异常葡萄糖代谢，因此，治疗第3天-第14天后，3组SUVmax值较治疗前（第0天）明显升高。鉴于炎症反应的干扰，^18^F-FDG/PET显像不能精确反映高剂量（370 MBq）粒子内照射治疗后肿瘤组织的变化。

治疗组病理学切片显示，近粒子处肿瘤细胞变性坏死，提示^32^P-CP-PLLA粒子持续照射可直接杀伤肿瘤细胞。远离粒子处虽可见存活肿瘤细胞，但凋亡基因如*bcl-2*和*bax*的表达明显异于对照组。*bcl-2*为抗凋亡基因，其下调有利于降低肿瘤细胞对放化疗的抗性，利于诱导细胞凋亡；*bax*为凋亡基因，其表达增高可抑制bcl-2的功能，促进凋亡，有利于肿瘤细胞死亡并可抑制肿瘤发生发展。bcl-2和bax可形成异二聚体，两者的比例与凋亡密切相关^[18]^。研究结果表明^32^P-CP-PLLA粒子可通过电离辐射诱导*bcl-2*和*bax*基因参与VX2移植瘤细胞凋亡过程的调控，从而抑制肿瘤生长，表现为bcl-2/bax比值明显下调。治疗第14天后，凋亡基因如*bcl-2*和*bax*的表达强度与粒子的放射性剂量明显相关。

本研究表明，^32^P-CP-PLLA粒子明显抑制VX2肿瘤的糖代谢，促进肿瘤细胞凋亡。由于VX2肿瘤和临床肿瘤的生物学特性并不完全相同，^32^P-CP-PLLA粒子内照射治疗的效果有待进一步研究。
